# The burden of road traffic crashes, injuries and deaths in Africa: a systematic review and meta-analysis

**DOI:** 10.2471/BLT.15.163121

**Published:** 2016-04-21

**Authors:** Davies Adeloye, Jacqueline Y Thompson, Moses A Akanbi, Dominic Azuh, Victoria Samuel, Nicholas Omoregbe, Charles K Ayo

**Affiliations:** a Demography and Social Statistics and the e-Health Research Cluster, Covenant University, Canaan land, PMB 1023, Ota, Ogun State, Nigeria.; b Nuffield Department of Orthopaedics, Rheumatology and Musculoskeletal Sciences, University of Oxford, Oxford, England.; c Department of Computer and Information Sciences and the e-Health Research Cluster, Covenant University, Ota, Nigeria.

## Abstract

**Objective:**

To estimate the burden of road traffic injuries and deaths for all road users and among different road user groups in Africa.

**Methods:**

We searched MEDLINE, EMBASE, Global Health, Google Scholar, websites of African road safety agencies and organizations for registry- and population-based studies and reports on road traffic injury and death estimates in Africa, published between 1980 and 2015. Available data for all road users and by road user group were extracted and analysed. We conducted a random-effects meta-analysis and estimated pooled rates of road traffic injuries and deaths.

**Findings:**

We identified 39 studies from 15 African countries. The estimated pooled rate for road traffic injury was 65.2 per 100 000 population (95% confidence interval, CI: 60.8–69.5) and the death rate was 16.6 per 100 000 population (95% CI: 15.2–18.0). Road traffic injury rates increased from 40.7 per 100 000 population in the 1990s to 92.9 per 100 000 population between 2010 and 2015, while death rates decreased from 19.9 per 100 000 population in the 1990s to 9.3 per 100 000 population between 2010 and 2015. The highest road traffic death rate was among motorized four-wheeler occupants at 5.9 per 100 000 population (95% CI: 4.4–7.4), closely followed by pedestrians at 3.4 per 100 000 population (95% CI: 2.5–4.2).

**Conclusion:**

The burden of road traffic injury and death is high in Africa. Since registry-based reports underestimate the burden, a systematic collation of road traffic injury and death data is needed to determine the true burden.

## Résumé

**Objectif:**

Estimer l'incidence des traumatismes et des décès dus à des accidents de la route pour toute la population des usagers et pour les différents groupes d'usagers de la route, en Afrique.

**Méthodes:**

Nous avons fait des recherches dans MEDLINE, EMBASE, Global Health, Google Scholar et sur des sites Internet d'organisations et d'agences de sécurité routière africaines, pour trouver des rapports et des études réalisées en population ou à partir de registres, concernant l'estimation des traumatismes et des décès dus à des accidents de la route en Afrique, publiés entre 1980 et 2015. Les données obtenues pour l'ensemble des usagers de la route et pour chaque groupe d'usagers ont été extraites et analysées. Nous avons effectué une méta-analyse à effets aléatoires et avons estimé les taux combinés des traumatismes et des décès dus à des accidents de la route.

**Résultats:**

Nous avons identifié 39 études, de 15 pays africains. Le taux combiné de traumatismes dus à des accidents de la route a été estimé à 65,2 pour 100 000 habitants (intervalle de confiance -IC- de 95%: 60,8–69,5) et le taux de décès a été estimé à 16,6 pour 100 000 habitants (IC de 95%: 15,2-18,0). Le taux de traumatismes dus à des accidents de la route a augmenté de 40,7 pour 100 000 habitants dans les années 1990 à 92,2 pour 100 000 habitants pour la période entre 2010 et 2015, tandis que le taux de décès est passé de 19,9 pour 100 000 habitants dans les années 1990 à 9,3 pour 100 000 habitants entre 2010 et 2015. Le taux le plus élevé de tués sur les routes correspond aux occupants de véhicules motorisés à quatre roues, avec 5,9 victimes de la route pour 100 000 habitants (IC de 95 %: 4,4–7,4), suivis de près par les piétons, avec 3,4 décès pour 100 000 habitants (IC de 95%: 2,5-4,2).

**Conclusion:**

L'incidence des traumatismes et des décès dus à des accidents de la route est élevée en Afrique. Étant donné que les rapports fondés sur des registres sous-estiment les chiffres réels, il est nécessaire de procéder à un enregistrement systématique des données sur les traumatismes et les décès dus à des accidents de la route pour pouvoir déterminer leur véritable incidence.

## Resumen

**Objetivo:**

Estimar la tasa de traumatismos y muertes por accidentes de tráfico para todos los usuarios de las carreteras y entre los distintos grupos de usuarios de las carreteras en África.

**Métodos:**

Se realizaron búsquedas en MEDLINE, EMBASE, Global Health, Google Scholar y sitios web de agencias y organizaciones de seguridad vial africanas para encontrar estudios e informes basados en la población y en los registros sobre estimaciones de traumatismos y muertes por accidentes de tráfico en África publicados entre 1980 y 2015. Se extrajeron y analizaron los datos disponibles para todos los usuarios de las carreteras y por grupo de usuarios de las carreteras. Se realizó un metaanálisis de efectos aleatorios y se estimaron tasas agrupadas de traumatismos y muertes por accidentes de tráfico.

**Resultados:**

Se identificaron 39 estudios de 15 países africanos. La tasa agrupada estimada de traumatismos por accidentes de tráfico fue de 65,2 por cada 100 000 habitantes (intervalo de confianza, IC, del 95%: 60,8–69,5) y la tasa de muertes fue de 16,6 por cada 100 000 habitantes (IC del 95%: 15,2–18,0). Las tasas de traumatismos por accidentes de tráfico aumentaron de 40,7 por cada 100 000 habitantes en la década de 1990 a 92,9 por cada 100 000 habitantes entre 2010 y 2015, mientras que las tasas de muertes se redujeron de 19,9 por cada 100 000 habitantes en la década de 1990 a 9,3 por cada 100 000 habitantes entre 2010 y 2015. La mayor tasa de muertes por accidentes de tráfico se encontró entre los ocupantes de vehículos motorizados de cuatro ruedas, con un 5,9 por cada 100 000 habitantes (IC del 95%: 4,4–7,4), seguida muy de cerca por los peatones, con un 3,4 por cada 100 000 habitantes (IC del 95%: 2,5–4,2).

**Conclusión:**

En África, la tasa de traumatismos y muertes por accidentes de tráfico es alta. Puesto que los informes basados en registros infravaloran dicha tasa, es necesario un cotejo sistemático de datos de traumatismos y muertes por accidentes de tráfico para determinar la tasa real.

## ملخص

**الغرض:**

تقدير عبء الإصابات وحالات الوفاة الناجمة عن حوادث المرور فيما يتعلق بمستخدمي الطرق في مجملهم وضمن المجموعات المختلفة لمستخدمي الطرق في أفريقيا.

**الطريقة:**

بحثنا في قواعد معطيات MEDLINE، وEMBASE، وGlobal Health، وGoogle Scholar، والمواقع الإلكترونية للهيئات المعنية بالحفاظ على السلامة على الطرق عن الدراسات القائمة على السجلات وقطاع السكان وكذلك التقارير التي تناولت تقديرات للإصابات وحالات الوفاة الناجمة عن حوادث المرور في أفريقيا والتي تم نشرها في الفترة بين عامي 1980 و2015. وتم استخلاص وتحليل البيانات المتاحة فيما يتعلق بمستخدمي الطرق في مجملهم والمصنفة حسب مجموعة مستخدمي الطرق. استخدمنا نموذج الآثار العشوائية للتحليل التلوي وقدّرنا المعدلات المجمعة للإصابات وحالات الوفاة الناجمة عن حوادث المرور.

**النتائج:**

حددنا 39 دراسة مستمدة من 15 دولة أفريقية. وبلغ المعدل المجمع للإصابات الناجمة عن حوادث المرور حسب التقديرات 65.2 لكل شريحة سكانية يبلغ عدد أفرادها 10,000 نسمة (بنسبة أرجحية مقدارها 95%: 60.8 – 69.5) وبلغ معدل الوفيات 16.6 لكل شريحة سكانية يبلغ عدد أفرادها 100,000 نسمة (بنسبة أرجحية مقدارها ‏95%: 15.2–18.0). وارتفعت معدلات الإصابة الناجمة عن حوادث الطرق من 40.7 لكل شريحة سكانية يبلغ عدد أفرادها 100,000 نسمة في التسعينيات من القرن العشرين لتصل إلى 92.9 لكل شريحة سكانية يبلغ عدد أفرادها 100,000 نسمة في الفترة بين 2010 و2015، بينما انخفضت معدلات الوفاة من 19.9 لكل شريحة سكانية يبلغ عدد أفرادها 100,000 نسمة في التسعينيات من القرن العشرين لتبلغ 9.3 لكل شريحة سكانية يبلغ عدد أفرادها 100,000 نسمة في الفترة بين عامي 2010 و2015. وكان أعلى معدل للوفيات الناجمة عن حوادث المرور لمستخدمي المركبات ذات الأربع عجلات المزودة بمحرك، حيث بلغ 5.9 لكل شريحة سكانية يبلغ عدد أفرادها 100,000 نسمة (بنسبة أرجحية مقدارها 95‏%: 4.4 – 7.4)، ويليهم بفارق صغير المشاة بمعدل يبلغ 3.4 لكل شريحة سكانية يبلغ عدد أفرادها 100,000 نسمة (بنسبة أرجحية مقدارها 95‏%: 2.5–4.2).

**الاستنتاج:**

إن عبء الإصابة وحالات الوفاة الناجمة عن حوادث الطرق مرتفع في أفريقيا. ونظرًا للدور الذي تلعبه التقارير القائمة على السجلات في خفض تقديرات العبء عن معدلاتها الحقيقية، فإن الأمر يقتضي تجميع البيانات المتعلقة بالإصابة وحالات الوفاة الناجمة عن حوادث المرور ومقارنتها بطريقة منهجية لتحديد العبء الحقيقي.

## 摘要

**目的:**

旨在为非洲所有的道路用户以及不同道路用户人群评估道路交通伤害和死亡的负担。

**方法:**

我们检索了联机医学文献分析和检索系统 (MEDLINE)、荷兰医学文摘数据库 (EMBASE)、全球健康 (Global Health)、谷歌学术 (Google Scholar) 和非洲道路安全机构和组织的网站，收集了 1980 年至 2015 年期间发布的关于非洲道路交通伤害和死亡估计的基于登记信息和人群的研究和报告。同时，摘取了针对所有道路用户以及道路用户人群提供的现有数据，并对其进行了分析。我们开展了随机效应元分析，并且评估了道路交通伤害和死亡的总发生率。

**结果:**

我们从 15 个非洲国家确定了 39 项研究。 道路交通伤害的总发生率预计为每 10 万人中 65.2 例（95% 置信区间，CI： 60.8–69.5）死亡率为每 10 万人中 16.6 例（95% CI：15.2–18.0）。与 20 世纪 90 年代相比，在 2010 年至 2015 年期间，道路交通伤害率从每 10 万人中 40.7 例上升到每 10 万人中 92.9 例，同期，死亡率从每 10 万人中 19.9 例减少到每 10 万人中 9.3 例。道路交通死亡发生率最高的人群是四轮机动车车主，为每 10 万人中 5.9 例 (95% CI: 4.4–7.4)，紧随其后的是行人，为每 10 万人中 3.4 例 (95% CI: 2.5–4.2)。

**结论:**

非洲道路交通事故和伤亡负担很重。由于基于登记信息的报告会低估负担程度，因此需要对道路交通伤害和死亡数据进行系统整理，以确定真实的负担程度。

## Резюме

**Цель:**

Оценить бремя травматизма и смертности в результате дорожно-транспортных происшествий для всех участников дорожного движения и для различных групп участников дорожного движения в Африке.

**Методы:**

Был выполнен поиск в базах данных MEDLINE, EMBASE, Global Health, системе Google Scholar, на веб-сайтах африканских учреждений по обеспечению безопасности дорожного движения и организаций на предмет реестровых и популяционных исследований травматизма и смертности в результате дорожно-транспортных происшествий в Африке и отчетов по ним, опубликованных в период между 1980 и 2015 годами. Доступные данные по всем участникам дорожного движения и по их отдельным группам были извлечены и проанализированы. С помощью метаанализа с использованием модели случайных эффектов были рассчитаны объединенные показатели травматизма и смертности в результате дорожно-транспортных происшествий.

**Результаты:**

Было выявлено 39 исследований из 15 стран Африки. Объединенный расчетный показатель дорожно-транспортного травматизма составил 65,2 случая на 100 000 жителей (95%-й доверительный интервал, ДИ: 60,8–69,5), а показатель смертности составил 16,6 случая на 100 000 жителей (95%-й ДИ: 15,2–18,0). Показатели травматизма в результате дорожно-транспортных происшествий увеличились с 40,7 случая на 100 000 жителей в 1990-х годах до 92,9 случая на 100 000 жителей в период с 2010 по 2015 год, а показатели смертности снизились с 19,9 случая на 100 000 жителей в 1990-х годах до 9,3 случая на 100 000 жителей в период с 2010 по 2015 год. Наибольший показатель смертности в результате дорожно-транспортных происшествий наблюдался среди пассажиров моторных четырехколесных транспортных средств и составлял 5,9 случая на 100 000 жителей (95%-й ДИ: 4,4–7,4); ненамного меньший показатель наблюдался среди пешеходов и составлял 3,4 случая на 100 000 жителей (95%-й ДИ: 2,5–4,2).

**Вывод:**

В Африке существует высокое бремя травматизма и смертности в результате дорожно-транспортных происшествий. Поскольку показатели бремени, полученные в результате реестровых исследований, занижены, для определения истинного бремени требуется систематизация данных по травматизму и смертности в результате дорожно-транспортных происшествий.

## Introduction

Road traffic injuries are among the leading causes of death and life-long disability globally.^1^ The World Health Organization (WHO) reports that about 1.24 million people die annually on the world’s roads, with 20–50 million sustaining non-fatal injuries.^1^^,^^2^ Globally, road traffic injuries are reported as the leading cause of death among young people aged 15–29 years and are among the top three causes of mortality among people aged 15–44 years.^1^ The Institute for Health Metrics and Evaluation (IHME) estimated about 907 900, 1.3 million and 1.4 million deaths from road traffic injuries in 1990, 2010 and 2013, respectively.^3^

In Africa, the number of road traffic injuries and deaths have been increasing over the last three decades.^4^ According to the 2015* Global status report on road safety*, the WHO African Region had the highest rate of fatalities from road traffic injuries worldwide at 26.6 per 100 000 population for the year 2013.^1^^,^^2^ In 2013, over 85% of all deaths and 90% of disability adjusted life years (DALYs) lost from road traffic injuries occurred in low- and middle-income countries, which have only 47% of the world’s registered vehicles.^2^^,^^3^ The increased burden from road traffic injuries and deaths is partly due to economic development, which has led to an increased number of vehicles on the road.^5^^,^^6^ Given that air and rail transport are either expensive or unavailable in many African countries, the only widely available and affordable means of mobility in the region is road transport.^1^^,^^2^^,^^7^ However, the road infrastructure has not improved to the same level to accommodate the increased number of commuters and ensure their safety and as such many people are exposed daily to an unsafe road environment.^1^^,^^4^

The 2009* Global status report on road safety* presented the first modelled regional estimate of a road traffic death rate, which was used to statistically address the underreporting of road traffic deaths by countries with an unreliable death registration system.^5^ In the 2009 report, Africa had the highest modelled fatality rate at 32.2 per 100 000 population, in contrast to the reported fatality rate of 7.2 per 100 000 population.^5^The low reported death rate reflects the problem of missing data due to non-availability of road traffic data systems, which has a direct impact on health planning including prehospital and emergency care and other responses by government agencies.

This study aimed to review existing literature on published studies, registry-based reports and unpublished articles on the burden of road traffic injuries and deaths in the African continent to generate a continent-wide estimate of road traffic injuries and deaths for all road users and by road user type (pedestrians, motorized four-wheeler occupants, motorized two–three wheeler users and cyclists).

## Methods

**Fig. 1:**
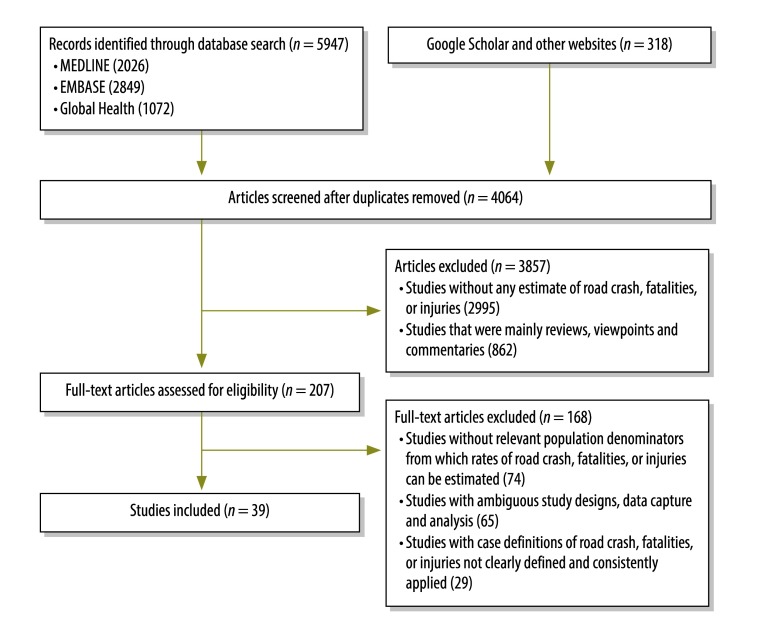
Selection of studies for the review on road traffic crashes, injuries and deaths in Africa, 1980–2015

We searched MEDLINE, EMBASE, Global Health, Google Scholar, websites of road safety agencies and relevant organizations within Africa for articles published between 1980 and 2015 (Fig. 1). The search strategy and terms are presented in Box 1 (available at: http://www.who.int/bulletin/volumes/94/7/15-163121). There was no language restriction.

Box 1 **Search strategy of published studies on the burden of road traffic crashes, injuries and deaths in Africa.**

1. africa/ or exp africa, northern/ or exp algeria/ or exp egypt/ or exp libya/ or exp morocco/ or exp tunisia/ or exp “africa south of the sahara”/ or exp africa, central/ or exp cameroon/ or exp central african republic/ or exp chad/ or exp congo/ or exp “democratic republic of the congo”/ or exp equatorial guinea/ or exp gabon/ or exp africa, eastern/ or exp burundi/ or exp djibouti/ or exp eritrea/ or exp ethiopia/ or exp kenya/ or exp rwanda/ or exp somalia/ or exp sudan/ or exp tanzania/ or exp uganda/ or exp africa, southern/ or exp angola/ or exp botswana/ or exp lesotho/ or exp malawi/ or exp mozambique/ or exp namibia/ or exp south africa/ or exp swaziland/ or exp zambia/ or exp zimbabwe/ or exp africa, western/ or exp benin/ or exp burkina faso/ or exp cape verde/ or exp cote d'ivoire/ or exp gambia/ or exp ghana/ or exp guinea/ or exp guinea-bissau/ or exp liberia/ or exp mali/ or exp mauritania/ or exp niger/ or exp nigeria/ or exp senegal/ or exp sierra leone/ or exp togo/

2. exp vital statistics/ or exp incidence

3. (incidence* or prevalence* or morbidity or mortality).tw.

4. (disease adj3 burden).tw.

5. exp “cost of illness”/

6. exp quality-adjusted life years/

7. QALY.tw.

8. Disability adjusted life years.mp.

9. (initial adj2 burden).tw.

10. exp risk factors/

11. 2 or 3 or 4 or 5 or 6 or 7 or 8 or 9 or 10

12. road traffic accident*.mp. [mp = title, abstract, original title, name of substance word, subject heading word, protocol supplementary concept, rare disease supplementary concept, unique identifier]

13. RTAs.mp. [mp = title, abstract, original title, name of substance word, subject heading word, protocol supplementary concept, rare disease supplementary concept, unique identifier]

14. road traffic injur*.mp. [mp = title, abstract, original title, name of substance word, subject heading word, protocol supplementary concept, rare disease supplementary concept, unique identifier]

15. traffic crash*.mp. [mp = title, abstract, original title, name of substance word, subject heading word, protocol supplementary concept, rare disease supplementary concept, unique identifier]

16. exp Accidents, Traffic/

17. exp air bags/ or exp child restraint systems/ or exp seat belts/

18. exp motor vehicles/ or exp automobiles/ or exp motorcycles/

19. 12 or 13 or 14 or 15 or 16 or 17 or 18

20. 1 and 11 and 19

21. limit 20 to (yr = ”1980 –Current

### Eligibility criteria

We included a study in the review if it met the following criteria: (i) conducted between 1980 and 2015 and that the study was done in an African country; (ii) clearly referred to road traffic crashes, injuries or deaths; (iii) referred data came from a population- or registry-based data system; (iv) registry-based hospital data with the underlying cause of death data coded in the International Classification of Disease and Related Health Problems, 10th revision (ICD-10), with codes V01–V89; (v) directly attempted to estimate the number or rate of road traffic crashes, injuries or deaths in a particular African country or the region as a whole; or (vi) provided any other information (e.g. response time, first responders) that may further help to understand the burden and determinants of road traffic crashes and policy response in the African region.

We excluded studies if they: (i) referred to deaths by other means of transportation including water, air and other unspecified transport means; (ii) were mainly reviews, viewpoints and commentaries; (iii) did not have a clearly defined study design, data capture and analysis method; and (iv) had not clearly defined and consistently applied a case definition of a road traffic crash, injury or fatality.

For this study, a crash is defined as a road traffic collision that resulted in an injury or fatality. Injury refers to non-fatal cases from a road traffic crash.^5^ Death is defined as a road traffic crash in which one or more persons involved in the crash died immediately or within 30 days of the crash.^2^ For non-fatal injuries, the case definition ranges from minor injuries with disabilities of short duration, to severe injuries with lifelong disabilities.

### Quality assessment

For each full text accessed, we checked if the study method had flaws in the design and execution. For the registry-based studies, we examined the study design, completeness, the appropriateness of statistical and analytical methods employed and if the limitations were explicitly stated. For each study, we assessed if the reported sample size or study population was appropriate to provide a representative estimate and if the heterogeneities within and between population groups undermine the pooled estimates. Studies not meeting this quality assessment were excluded.

### Data collection process

Available data from all selected studies were extracted twice, compiled and stored in a spreadsheet. For each study, data on the country, study period, study design, sample size, mean age and case definitions were extracted (Table 1). Reported road traffic crash, injury and death data for the overall study population and for the various categories of road users were extracted. The data were grouped by study setting and year of study, with corresponding age and sex categories.

**Table 1:** Studies on burden of road traffic crashes, injuries and deaths in Africa, as identified through a systematic review of the literature, 1980–2015

Reference	Study period	Country, study setting	Study design	Study type	Source of data	Type of data	Case definition
Sobngwi-Tambekou^8^	2004–2007	Cameroon, Yaoundé-Douala	Retrospective study	Registry-based	Police records	Death	Deaths within 30 days of a crash
Twagirayezu^9^	2005	Rwanda, Kigali	Descriptive and cross-sectional	Registry-based	Hospital records	Death and injury	Deaths at the site of a road crash or injured patients presenting to a hospital
Abegaz^10^	2012–2013	Ethiopia, Addis-Ababa	Capture–recapture	Registry-based	Traffic reports	Death and injury	Injuries and deaths resulting from a road crash
Mekonnen^11^	2007–2011	Ethiopia, Amhara region	Retrospective and descriptive study	Registry-based	Police road crash records	Death and injury	Injuries and deaths resulting from a road crash
Bachani^12^	2004–2009	Kenya	Retrospective and observational	Registry-based	Police (Kenya traffic police); vital registration (National Vital Registration System)	Death	Deaths within 30 days of crash
Ngallaba^13^	2009–2012	United Republic of Tanzania, Mwanza	Retrospective design	Registry-based	Hospital ward register and case notes, medical record; police records	Death	Deaths at the site of road crash or injured patients presenting to a hospital
Zimmerman^14^	2011–2012	United Republic of Tanzania	Cross-sectional	Population-based	Resident population	Death	Deaths within 30 days of a crash
Museru^15^	1990–2000	United Republic of Tanzania	Retrospective and descriptive	Registry-based	Police records	Death	Deaths at crash scene and outside scene
Barengo^16^	1990–2001	United Republic of Tanzania, Dar es Salaam	Cross-sectional	Registry-based	Routine police record	Death and injury	Injuries and deaths resulting from road crash
Kilale^17^	1995–1996	United Republic of Tanzania, Kiluvya-Bwawani and Chalinze-Segera	Retrospective and descriptive	Registry-based	Police road traffic crash records from the Coast Region Traffic Office	Death	Death at crash scene and outside scene
Nakitto^18^	2004–2005	Uganda, Kawempe	Cohort	Population-based	35 primary schools followed for three academic school year terms	Death and injury	Road traffic accidents with injuries and deaths on the spot and within 30 days of a crash
Bezzaoucha^19^	1986	Algeria	Cohort	Population-based	Resident population	Death	Fatalities defined as deaths at crash scene or outside scene
Bodalal^20^	2010–2011	Libya, Benghazi	Retrospective	Registry-based	Hospital records	Death and injury	Deaths at the site of road crash, Injured patients presenting to hospital immediately, or delayed presentation of a previously stable patient with fresh complaints
Samuel^21^	2008–2009	Malawi	Capture–recapture	Registry-based	Police and hospital records	Death	Deaths within 30 days of crash
Romão^22^	1990–2000	Mozambique	Retrospective	Registry-based	Transport records (National Institute for Road Safety)	Death and injury	Injuries and deaths at the site of road crash
Olukoga^23^	2003	South Africa	Retrospective and descriptive	Registry-based	Transport records (Department of Transport, Pretoria)	Death	Deaths within 30 days of crash
Olukoga^24^	2002–2003	South Africa	Retrospective and descriptive	Registry-based	National Statistics Office (Durban municipality)	Death	Deaths within 30 days of crash
Meel^25^	1993–1999	South Africa	Retrospective	Registry-based	Mortuary (medico-legal autopsy records)	Death	Deaths within 30 days of crash
Lehohla^26^	2001–2006	South Africa	Retrospective	Registry-based	National Statistics Office	Death and injury	Injuries and deaths resulting from road crash
Hobday^27^	2007	South Africa, eThekwini	Retrospective	Registry-based	Transport records (eThekwini transport authority database)	Death and injury	Child pedestrian injuries and deaths resulting from road crash
Kyei^28^	2004–2008	South Africa, Limpopo	Retrospective	Registry-based	Transport management cooperation records	Death and injury	Injuries and deaths resulting from road crash
Meel^29^	1993–2004	South Africa, Mthatha	Retrospective	Registry-based	Mortuary (death records and autopsies from Mthatha and Nqgeleni magisterial districts)	Death	Deaths within 30 days of crash
Amonkou^30^	2001–2011	Cote d’Ivoire, Abidjan	Retrospective	Registry-based	Hospital records	Death and injury	Injuries and deaths resulting from road crash
Ackaah^31^	2005–2007	Ghana	Retrospective and descriptive	Registry-based	Traffic reports, hospital records	Death	Fatalities where one or more persons are killed as a result of a crash and where the death occurs within 30 days of the crash
Afukaar^32^	1994–1998	Ghana	Retrospective and descriptive	Registry-based	Reported traffic crash data	Death and injury	Death within 30 days, serious injuries (hospitalization for > 24hrs), slight injuries (hospitalization for < 24hrs)
Guerrero^33^	2009	Ghana	Survey	Population-based	Resident population	Death and injury	Fatalities within 30 days of crash
Mock^34^	1999	Ghana	Survey	Population-based	Resident population	Death and injury	Road crash with injuries and deaths
Aidoo^35^	2004–2010	Ghana	Retrospective	Registry-based	Traffic reports	Death and injury	Pedestrian deaths and injuries from hit-and-run cases
Kudebong^36^	2004–2008	Ghana, Bolgatanga	Retrospective	Registry-based	Traffic reports	Death and injury	Deaths and injuries resulting from motorcycle road crash
Mamady^37^	2011	Guinea	Retrospective	Registry-based	Health ministry information	Death	Deaths as recorded in the country’s death notification form and coded using International Classification of Diseases, ninth revision (ICD-9)
Ezenwa^38^	1980–1983	Nigeria	Retrospective	Registry-based	Police records from federal police headquarters	Death	Death at scene of accident and outside scene
Nigeria Federal Road Safety Corps^39^	2001–2013	Nigeria	Surveillance	Registry-based	Federal Road Safety Corps data	Death	Death at scene of accident and outside scene
Labinjo^40^	2009	Nigeria	Survey	Population-based	Resident population	Death	Deaths within 30 days of crash
Asogwa^41^	1980–1985	Nigeria	Retrospective	Registry-based	Police records	Death	Accidents resulting in deaths
Balogun^42^	1987–1990	Nigeria, Ile-Ife	Retrospective	Registry-based	Hospital records	Death and injury	Deaths at the site of road crash or injured patients presenting to hospital
Adeoye^43^	2006–2007	Nigeria, Ilorin	Surveillance	Registry-based	Hospital records	Death and injury	Deaths at the site of road crash or injured patients presenting to hospital
Adewole^44^	2001–2006	Nigeria, Lagos	Audit	Registry-based	Ambulance service records	Death and injury	Deaths and injuries resulting from road crash
Jinadu^45^	1980–1982	Nigeria, Oyo	Retrospective and descriptive	Registry-based	Information from health and transport ministries	Death and injury	Deaths and injuries resulting from road crash
Aganga^46^	1980	Nigeria, Zaria	Retrospective	Registry-based	Traffic reports	Death and injury	Deaths and injuries resulting from road crash

### Data analysis

All extracted data on road traffic crashes, injuries and deaths were converted to rates per 100 000 population. Studies were subdivided into population- and registry-based studies and analysed separately for all road users and by road user category. A random effects meta-analysis was conducted on extracted road traffic crash, injury and death rates. To give a better understanding of the data distribution and comparisons with the pooled estimates and the confidence intervals, we further presented the range, median and data points within each data set. All statistical analyses were done in Excel 2010 (Microsoft, Redmond, United States of America) and Stata version 13.1 (StataCorp. LP, College Station, United States of America).

## Results

The review identified 39 studies reporting on 15 African countries (Table 1). Six were population-based and the remaining 33 were registry-based studies. Two studies were from Ethiopia,^10^^,^^11^ six from Ghana,^31^^–^^36^ nine from Nigeria,^38^^–^^46^ seven from South Africa^23^^–^^29^ and five from the United Republic of Tanzania.^13^^–^^17^ The remaining 10 studies were from one of the following countries: Algeria,^19^ Cameroon,^8^ Cote d’Ivoire,^30^ Guinea,^37^ Kenya,^12^ Libya,^20^ Malawi,^21^ Mozambique,^22^ Rwanda^9^ and Uganda.^18^ More than half (22) of the studies were conducted after the year 2000. The study period ranged from one year to 12 years, with a mean of 4.5 years. The full data set is available from the corresponding author.

### Reported rates

From all registry-based studies, Nigeria recorded the highest and lowest total crash rate at 716.57 per 100 000 population and 2.9 per 100 000 population, in 1990 and 2011, respectively.^39^^,^^42^ Ethiopia recorded the highest death rate at 81.6 per 100 000 population in 2011,^11^ while the lowest death rate was recorded in Nigeria at 1.64 per 100 000 population in 2007.^43^

From the available population-based studies, Nigeria reported the highest number of road traffic injury and death rates at 4120 per 100 000 population and 160 per 100 000 population, respectively. The road traffic injury rate is the highest recorded in any single study in Africa. Algeria and Ghana also reported high road traffic injury rates at 700 and 938 per 100 000 population, respectively.^19^^,^^34^ Only six studies reported male and female road traffic crash estimates,^14^^,^^19^^,^^21^^,^^22^^,^^29^^,^^31^ with Algeria and South Africa recording the highest number of casualties.

### Pooled rates

**Fig. 2:**
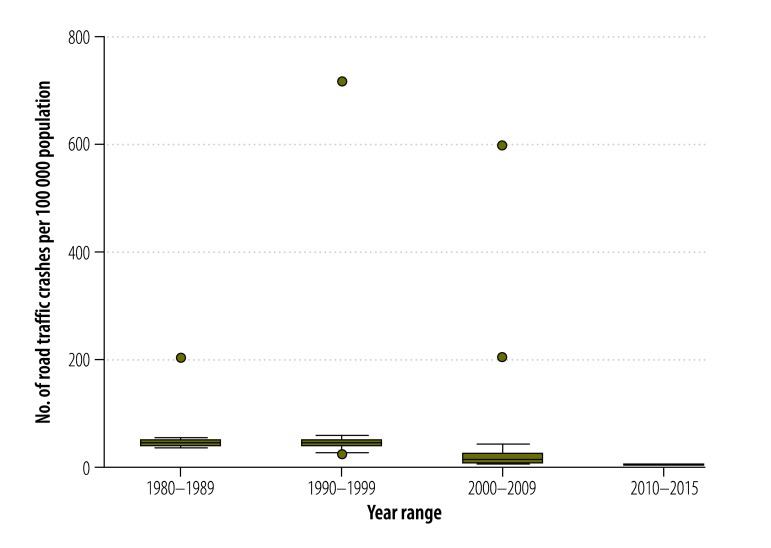
Pooled road traffic crash rate, Africa, 1980–2015

Note: In the box plot, the boxes represent the interquartile range of road traffic injury rates where the middle 50% (25–75%) of data are distributed; the bars represent road traffic injury rates outside the middle 50% (< 25% or > 75%); the dots represent specific road traffic injury rates which were a lot higher than normally observed over the study period (outliers) and the lower, middle and upper horizontal lines represent the minimum, median and maximum road traffic injury rates (excluding outliers), respectively.

**Fig. 3:**
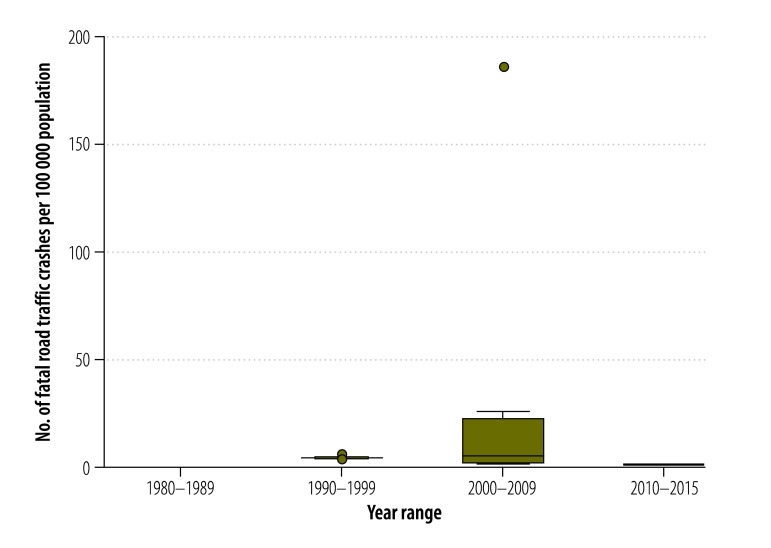
Pooled fatal road traffic crash rate, Africa, 1980–2015

Note: In the box plot, the boxes represent the interquartile range of road traffic injury rates where the middle 50% (25–75%) of data are distributed; the bars represent road traffic injury rates outside the middle 50% (< 25% or > 75%); the dots represent specific road traffic injury rates which were a lot higher than normally observed over the study period (outliers) and the lower, middle and upper horizontal lines represent the minimum, median and maximum road traffic injury rates (excluding outliers), respectively.

Table 2 presents the estimated pooled rates for the African continent. For total crashes, the pooled rate was 52.8 per 100 000 population, with the median at 39.7 per 100 000 population. The pooled fatal crash rate was estimated at 9.6 per 100 000 population with a median at 4.8 per 100 000 population. Pooled crash injury and death rates were estimated at 65.2 injuries and 16.6 deaths with medians of 38.9 injuries and 7.9 deaths per 100 000 population, respectively (Fig. 2 and Fig. 3).

**Table 2:** Pooled road traffic crash, injury and death estimates by road user type, African, 1980–2015

Road user type	Total crash rate^a^	Fatal crash rate^b^	Injury rate^c^	Death rate^d^
**All road users**				
Pooled rate (95% CI)	52.8 (49.0–56.6)	9.6 (8.6–10.7)	65.2 (60.8–69.5)	16.6 (15.2–18.0)
Median (range of estimates)	39.7 (2.9–716.6)	4.8 (0.7–186.1)	38.9 (8.1–491.8)	7.9 (1.6–81.6)
No. of data points	49	30	59	95
**Pedestrians **				
Pooled rate (95% CI)	–	–	10.8 (8.7–12.8)	3.4 (2.5–4.2)
Median (range of estimates)	–	–	9.14 (0.4–75.0)	2.2 (0.3–13.0)
No. of data points	–	–	28	21
**Four-wheelers**				
Pooled rate (95% CI)	–	–	37.2 (25.7–48.7)	5.9 (4.4–7.4)
Median (range of estimates)	–	–	26.6 (1.4–271.0)	2.7 (0.7–63.0)
No. of data points	–	–	19	23
**Two–three wheelers/cyclists**				
Pooled rate (95% CI)	–	–	16.1 (12.1–20.2)	1.3 (0.98–1.6)
Median (range of estimates)	–	–	5.8 (0.4–136.0)	0.9 (0.12–3.1)
No. of data points	–	–	19	22

CI: confidence interval.

^a^ Defined as number of all road traffic crashes (fatal and non-fatal) per 100 000 population.

^b^ Defined as number of fatal road traffic crashes per 100 000 population.

^c^ Defined as number of non-fatal road traffic injuries per 100 000 population.

^d^ Defined as number of fatal road traffic injuries per 100 000 population.

Note: There were no studies with crash rate and fatal crash rate to estimate for pedestrians, four-wheelers and two–three wheelers/cyclists.

**Fig. 4:**
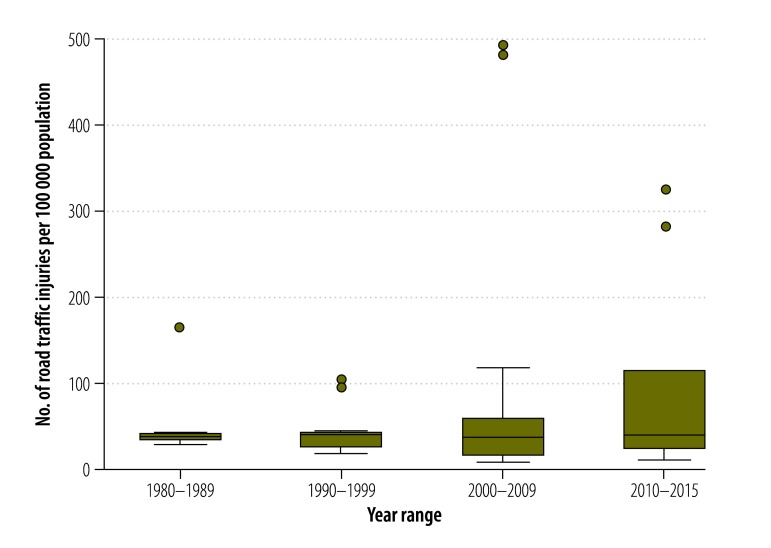
Pooled road traffic injury rate, Africa, 1980–2015

Note: In the box plot, the boxes represent the interquartile range of road traffic injury rates where the middle 50% (25–75%) of data are distributed; the bars represent road traffic injury rates outside the middle 50% (< 25% or > 75%); the dots represent specific road traffic injury rates which were a lot higher than normally observed over the study period (outliers) and the lower, middle and upper horizontal lines represent the minimum, median and maximum road traffic injury rates (excluding outliers), respectively.

**Fig. 5:**
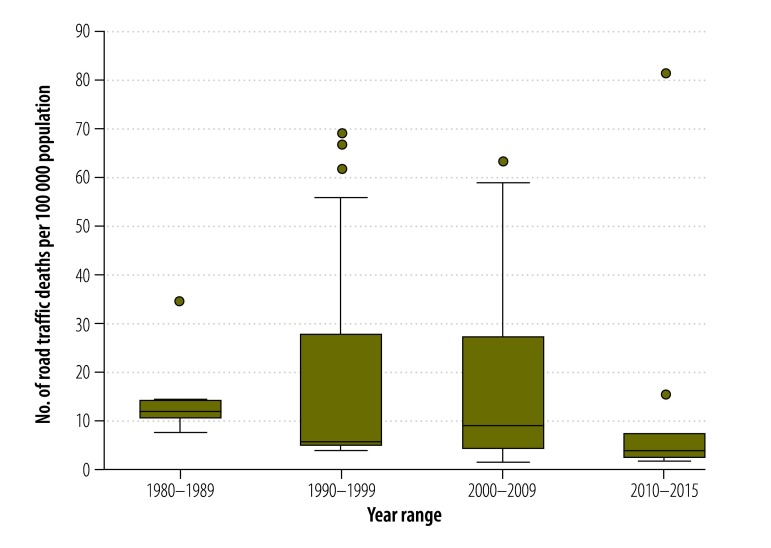
Pooled road traffic death rate, Africa, 1980–2015

Note: In the box plot, the boxes represent the interquartile range of road traffic injury rates where the middle 50% (25–75%) of data are distributed; the bars represent road traffic injury rates outside the middle 50% (< 25% or > 75%); the dots represent specific road traffic injury rates which were a lot higher than normally observed over the study period (outliers) and the lower, middle and upper horizontal lines represent the minimum, median and maximum road traffic injury rates (excluding outliers), respectively.

From 1990 to 2015, road traffic injury rates increased from 40.7 to 92.9 per 100 000 population (Table 3). In contrast, death rates decreased from 19.9 to 9.27 per 100 000 population (Fig. 4 and Fig. 5). Applying these figures and using the United Nations (UN) population estimates^47^ for the region, the pooled estimate came to 106 000 road traffic deaths and 1.1 million injuries in 2015, compared with 126 000 deaths and 260 000 injuries in 1990.

**Table 3:** Ten year pooled road traffic injury and death rate estimate, Africa, 1980–2015

Ten year range	Injury rate^a^ (95% CI)	Death rate^b^ (95% CI)
1980–1989	48.4 (44.5– 52.2)	12.6 (11.7–13.6)
1990–1999	40.7 (35.8–45.6)	19.9 (14.8–25.0)
2000–2009	75.6 (70.0–83.1)	16.5 (14.5–18.6)
2010–2015^c^	92.9 (84.8–101.0)	9.3 (8.2–10.3)

CI: confidence interval.

^a^ Defined as number of non-fatal road traffic injuries per 100 000 population.

^b^ Defined as number of fatal road traffic injuries per 100 000 population.

^c^ Covers only five years.

#### By road user category

From individual studies, road traffic death rates among pedestrians ranged from 0.26 per 100 000 population in Nigeria in 2007 to 13 per 100 000 population in South Africa in 2003.^43^^,^^24^ The death rate among motorized four-wheeler occupants was lowest in Nigeria in 2007 and highest in South Africa in 1999 at 0.74 and 63 per 100 000 population, respectively.^43^^,^^25^ A 2007 study from Cameroon reported the lowest road traffic death rate for motorized two–three wheeler occupants and cyclists and a 2012 study from the United Republic of Tanzania reported the highest, at 0.12 and 3.12 per 100 000 population, respectively.^8^^,^^13^ The pooled rates showed that motorized four-wheeler occupants had the highest road traffic death rate, closely followed by pedestrians. The pooled road traffic injury and death rates among pedestrians were 10.8 and 3.4 per 100 000 population, respectively. Among motorized four-wheeler occupants, the pooled road traffic injury and death rates were 37.2 and 5.9 per 100 000 population, respectively. Among motorized two–three wheeler occupants and cyclists, the pooled injury and death rates were 16.1 and 1.3 per 100 000 population, respectively (Table 2).

## Discussion

Our study reflects the difficulties that many experts have noted in describing the extent of road traffic crashes, injuries and deaths in Africa, for which modelling based on scarce and variable information, may not necessarily provide a reliable estimate.^48^ Moreover, registry-based reports may grossly underestimate the burden of road traffic crashes. Population-based studies consistently report a higher fatality rate.^19^^,^^34^^,^^40^ For example, a population-based survey conducted in Ghana in 1998 reported an injury rate of 940 per 100 000 population,^34^ while another registry-based study in the same country for the same year estimated 32 per 100 000 population.^32^ The Nigerian Federal Road Safety Corps estimated 3.7 deaths per 100 000 population for Nigeria in 2009.^39^ In contrast, a population-based study in the same country reported a higher estimate of 160 deaths per 100 000 population.^40^

The subgroup analysis showed that injury rates increased and death rates decreased between 1990 and 2015. A high road traffic injury number may reflect the effect of economic growth on the burden of road traffic injury in the region, which may be associated with increased travel and exposure to a hazardous traffic environment.^49^^,^^50^ However, death figures may be decreasing due to a relatively improving prehospital and emergency response system,^51^ as noted in Ghana, South Africa and Zambia.^52^^, ^^53^^,^^54^ It is important to note that many deaths may be missed or not recorded, as many of the road safety agencies tend to only record crashes, leaving the recording of deaths to health agencies.^55^^,^^56^

Our findings further revealed that the highest rates of casualties are among motorized four-wheeler occupants and pedestrians. A WHO report shows that 43% and 38% of road traffic deaths in the African Region occurred among motorized four-wheeler occupants and pedestrians, respectively.^2^ In Africa, most of these motorized four-wheeler occupants are passengers of commercial vehicles which is the commonly used means of transport.^4^ The high death rate among motorized four-wheeler occupants may also be due to the fact that crashes involving motorized four-wheeled vehicles are often recorded, while pedestrian crashes may be missed.^4^^,^^35^ However, we agree with some authors who have reported that pedestrians may be more affected in Africa due to bad road infrastructure, lack of pedestrian-friendly road signs, the way traffic is mixed with other road users and a general disregard for pedestrians by drivers.^1^

Meanwhile, a major challenge for the response to road crashes in Africa is the lack of reliable information and data that can inform an evidence-based public health response.^49^^,^^57^ Underreporting especially of vulnerable road users, poor or absent links between reporting agencies, exemptions from reporting, poor sampling techniques and varying case definitions have been indicated as limitations of reported data. The different rates of road traffic crash, injury and death reported in this study may be mostly related to surveillance system reporting errors and biases. In many African countries, there are no effective vital registration and active surveillance systems to capture the outcome of a road traffic crash^1^ and police data is the main source of traffic crash data.^1^^,^^2^ However, data from police sources tend to underreport injuries and deaths due to poor traffic police response and follow up on injured victims and varying traffic fatality definitions for real-time and chronologic data capture.^4^

Our study has the following limitations. Population-based studies on road traffic crashes in Africa, which would have been more reliable than registry-based studies, were not available. Population-based studies may have given insights on the extent of road users’ exposure to traffic risk, mode and frequency of road travel, distance travelled, number of road commuters and the conditions of the road. In the absence of such information, we have not based our estimates on an appropriate travel exposure denominator, thus limiting an understanding of the reasons behind the reported road traffic crash, injury and death rates and trends.

The available registry-based studies varied in their quality. They reported questionable values and trends and provided uncertain estimates. Lack of appropriate case definition for road traffic fatalities and incomplete breakdown of road traffic crash estimates by road user type were major limitations. Additionally, the non-fatal injury figures reported by the different studies varied with respect to severity and outcome. These variations could have affected our meta-analyses.

While we applied the UN population data for Africa to estimate rates where relevant national reference population data were unavailable, there were no comparable data to use for subnational studies. In addition, the data employed for this analysis were generated only from 15 countries, which is relatively small to accurately reflect the overall situation in the region. Hence, our estimates should be interpreted against these limitations.

In conclusion, our study suggests that the burden of road traffic injuries in Africa is high and there is an underestimation of road traffic fatalities. Improved road traffic injury surveillance across African countries may be useful in identifying relevant data gaps and developing contextually feasible prevention strategies in these settings.
